# Supplementary material for: DISSEQT—DIStribution-based modeling of SEQuence space Time dynamics
Source: Virus Evol. 2019 Aug 5;5(2):vez028. doi: 10.1093/ve/vez028 (PMC6680062; doi:10.1093/ve/vez028)
Supplement: vez028_Supplementary_Data [file vez028_supplementary_data.zip › SupplementaryFigures.pdf]

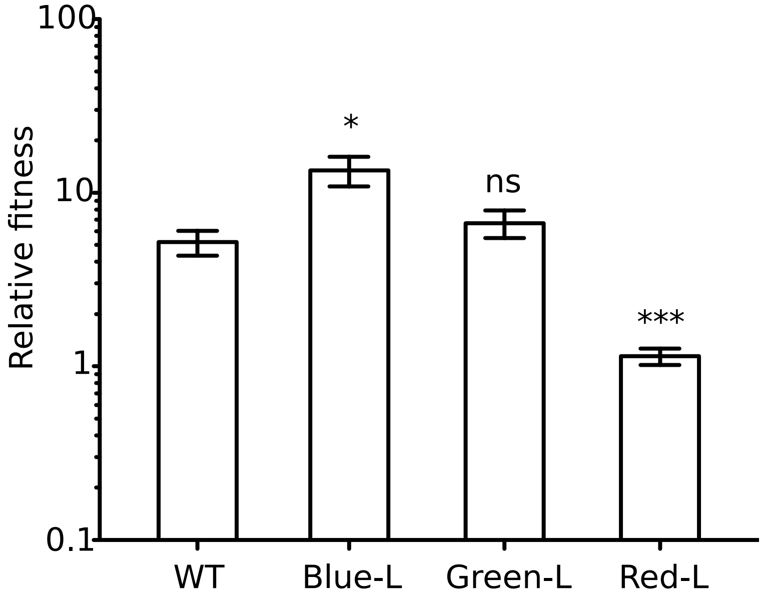

Supplementary Figure 1: Relative fitness by direct competition assay. Wild type (WT), Blue Lineage (Blue-L), Green Lineage (Green-L) and Red Lineage (Red-L). Mean and SEM are shown, $n = 6$, two-tailed unpaired t-test with Bonferroni correction. ns, not significant, $p = 0.334$; * $p = 0.013$; *** $p = 0.0008$.

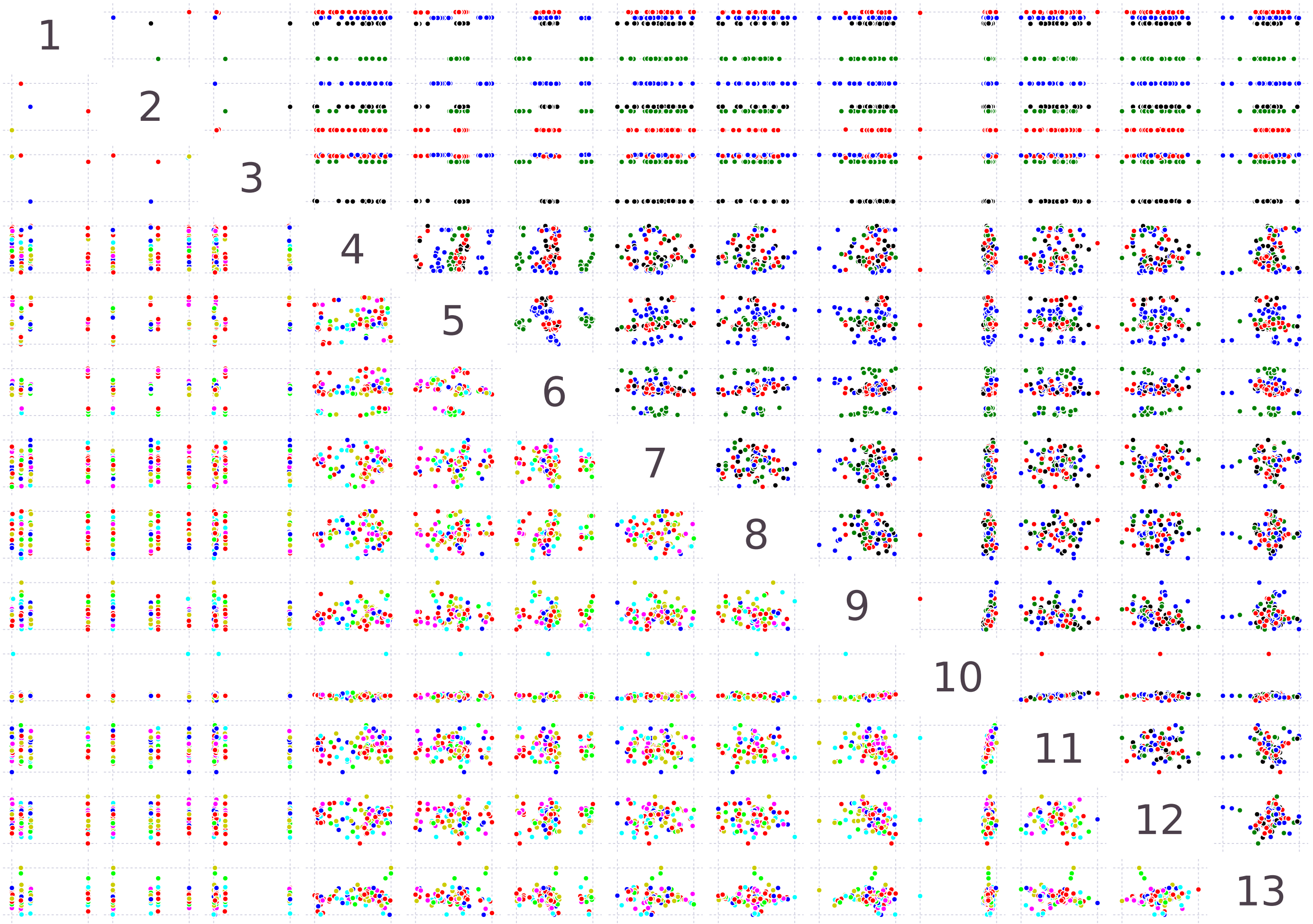

Supplementary Figure 2: Pairwise scatter plots showing the first 13 principal components in the analysis of the SynSyn data set plotted against each other, using consensus information only for the sequence space model. Plots above and below the diagonal are mirror images of each other. Each dot represents one viral population. Above the diagonal, samples are colored by lineage (Black: 1, Blue: 2, Green: 3, Red: 4) and below the diagonal, samples are colored by mutagen (Red: 5-fluorouracil, Light green: amiloride, Blue: 5-azacytidine, Yellow: Mn^{2+}, Cyan: ribavirin, Magenta: mock). All axes are rescaled to fill the plot area.

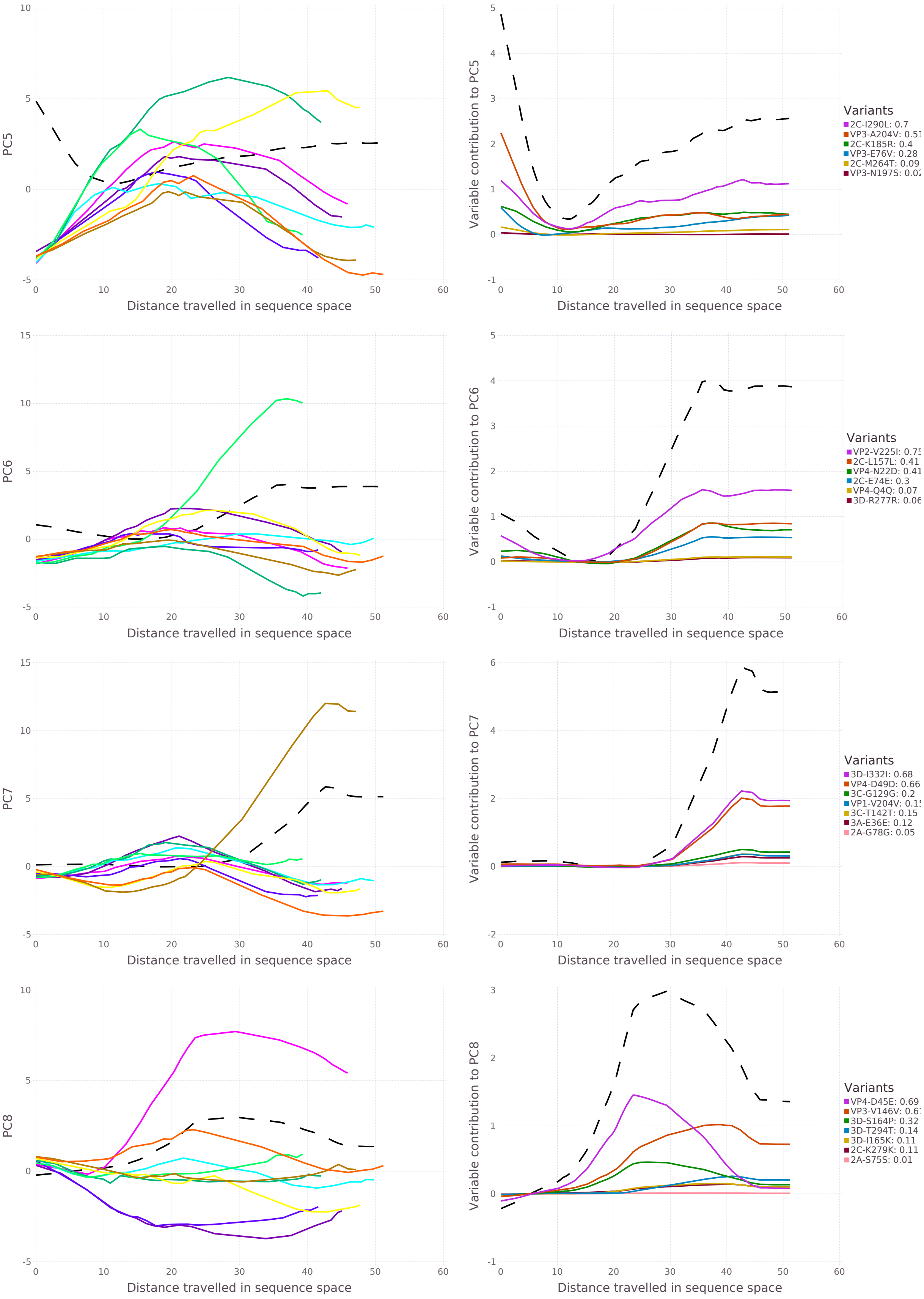

Supplementary Figure 3: **A.** Components 5–8. Left column: Principal components for replicates as a function of arc length. Right column: Variable contributions as a function of arc length. Both columns: The dotted black line shows the total contribution to σ_k at s.

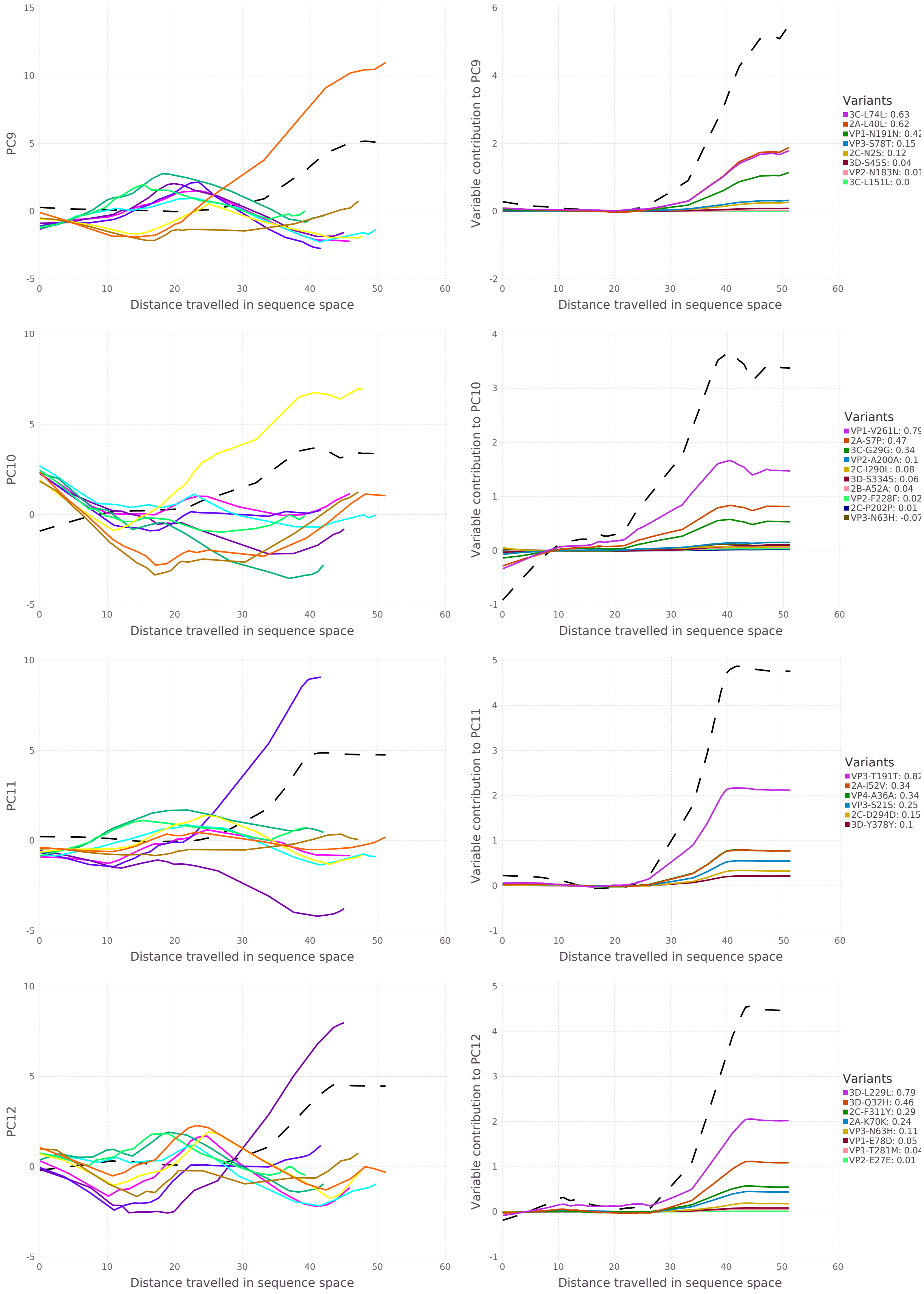

Supplementary Figure 3: **B.** Components 9–12. Left column: Principal components for replicates as a function of arc length. Right column: Variable contributions as a function of arc length. Both columns: The dotted black line shows the total contribution to σ_k at s.

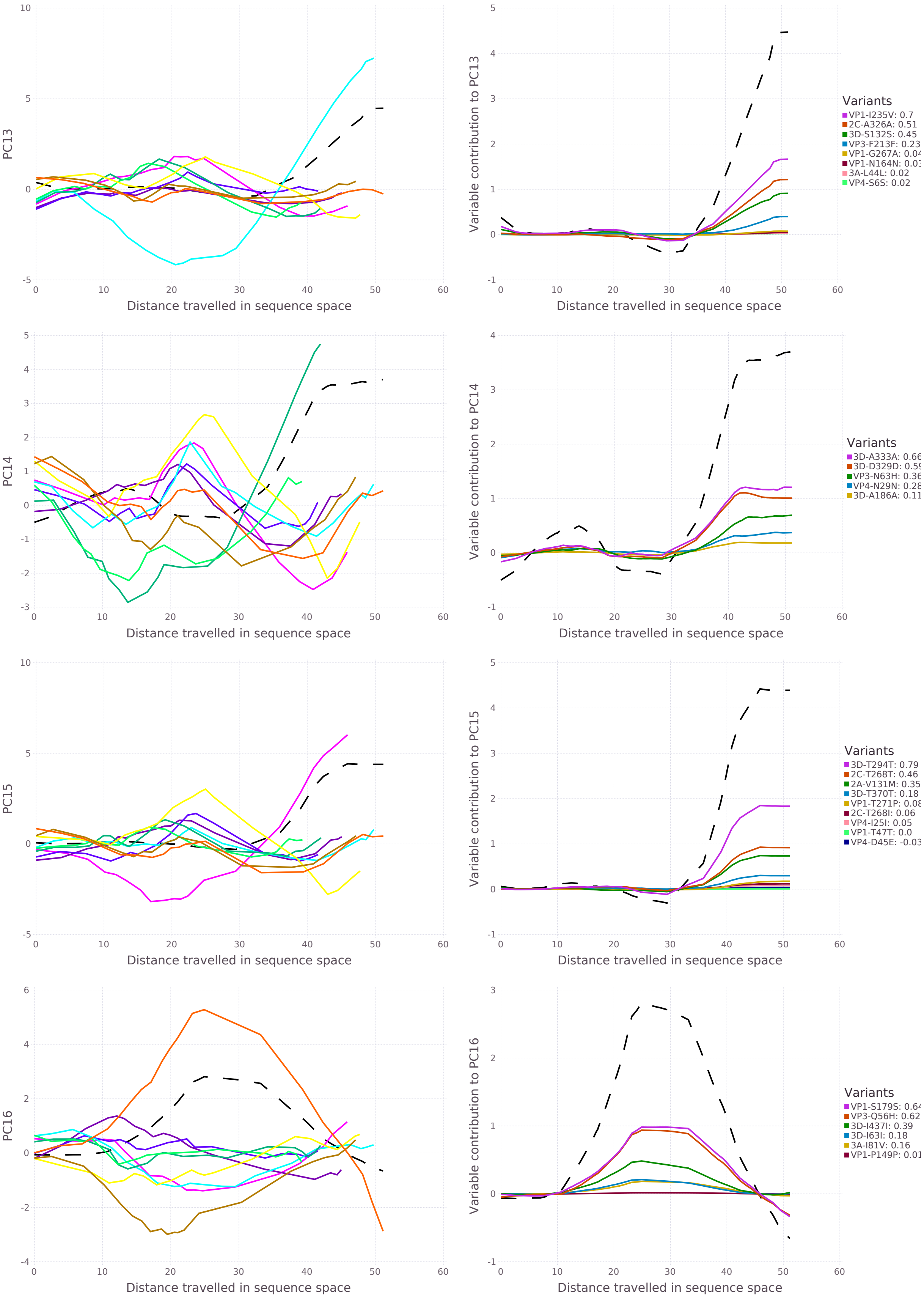

Supplementary Figure 3: **C.** Components 13–16. Left column: Principal components for replicates as a function of arc length. Right column: Variable contributions as a function of arc length. Both columns: The dotted black line shows the total contribution to σ_k at s.

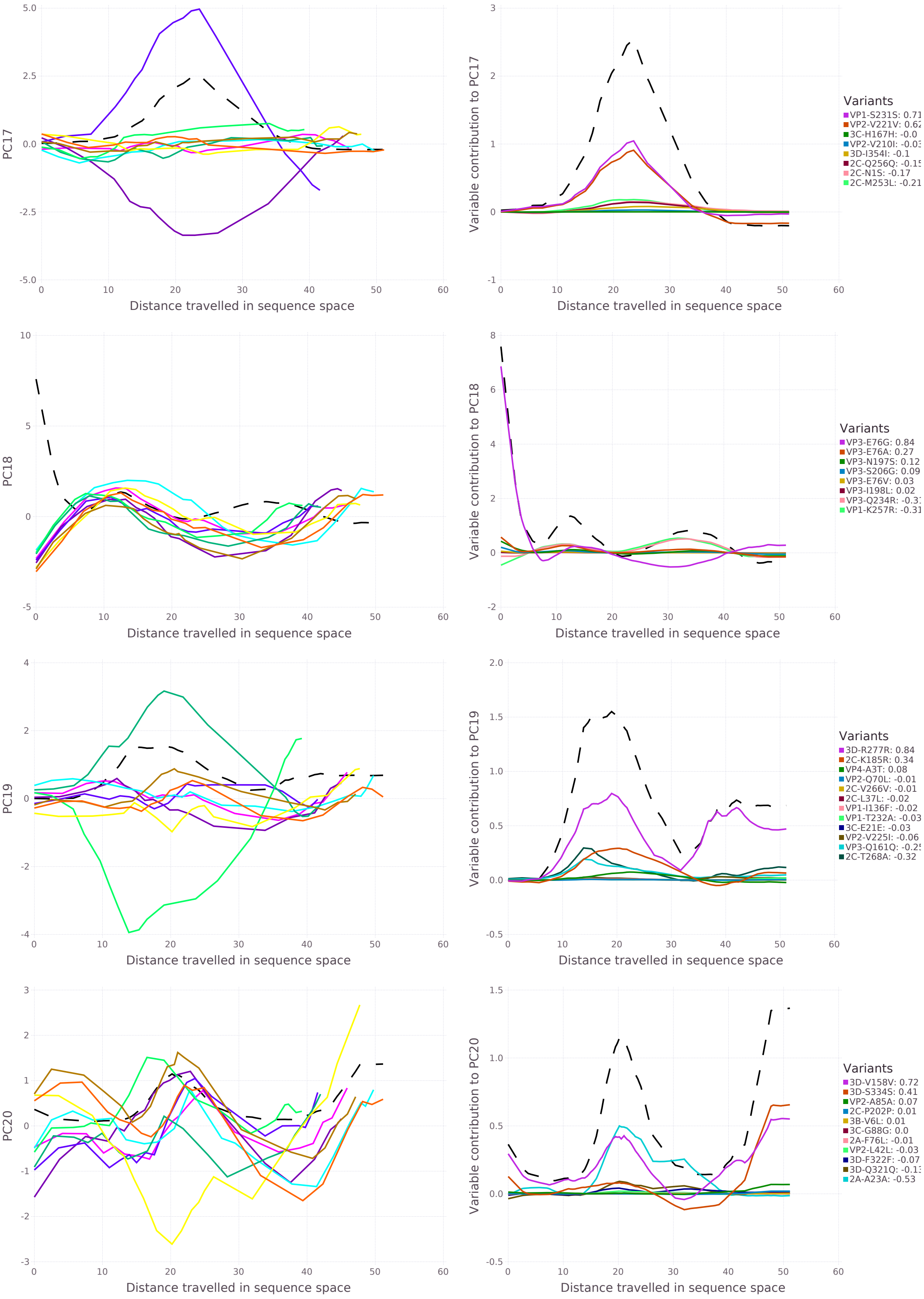

Supplementary Figure 3: **D.** Components 17–20. Left column: Principal components for replicates as a function of arc length. Right column: Variable contributions as a function of arc length. Both columns: The dotted black line shows the total contribution to σ_k at s.

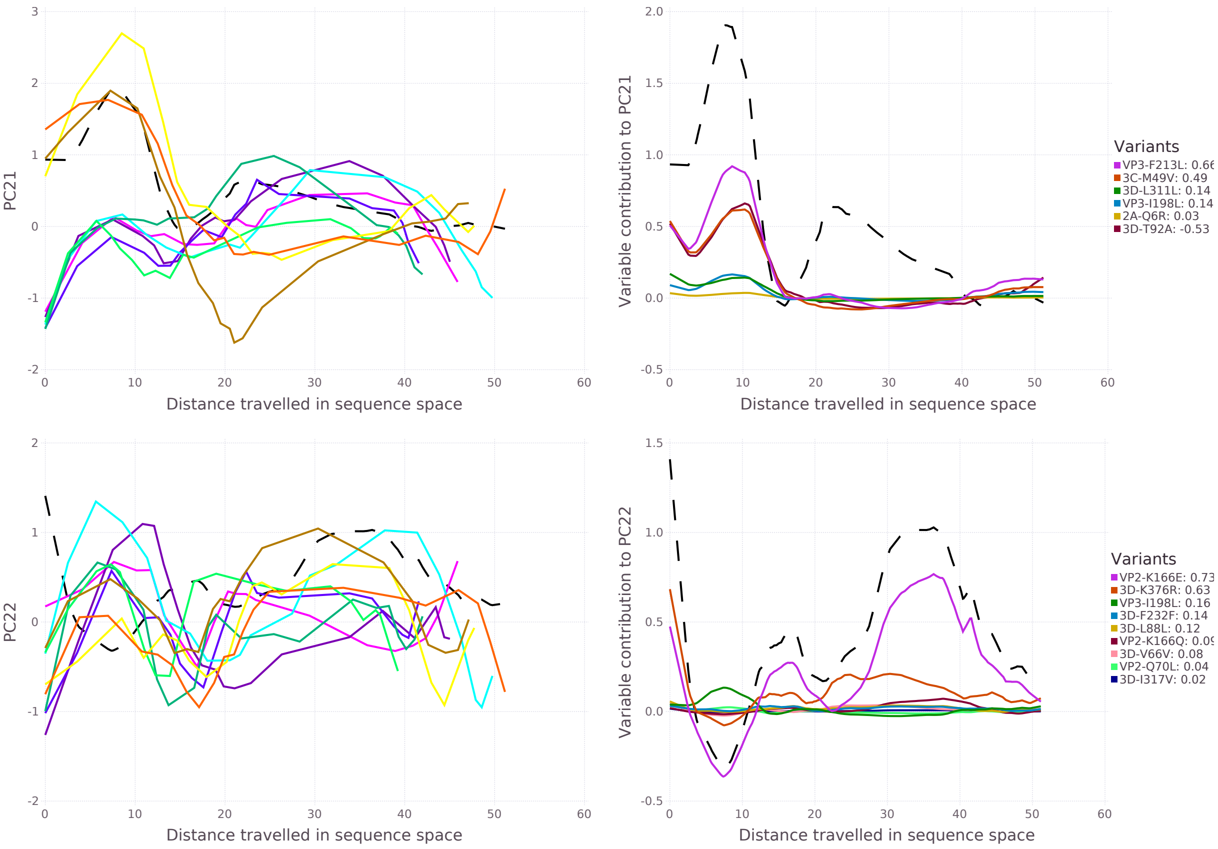

Supplementary Figure 3: **E.** Components 21–22. Left column: Principal components for replicates as a function of arc length. Right column: Variable contributions as a function of arc length. Both columns: The dotted black line shows the total contribution to σ_k at s.

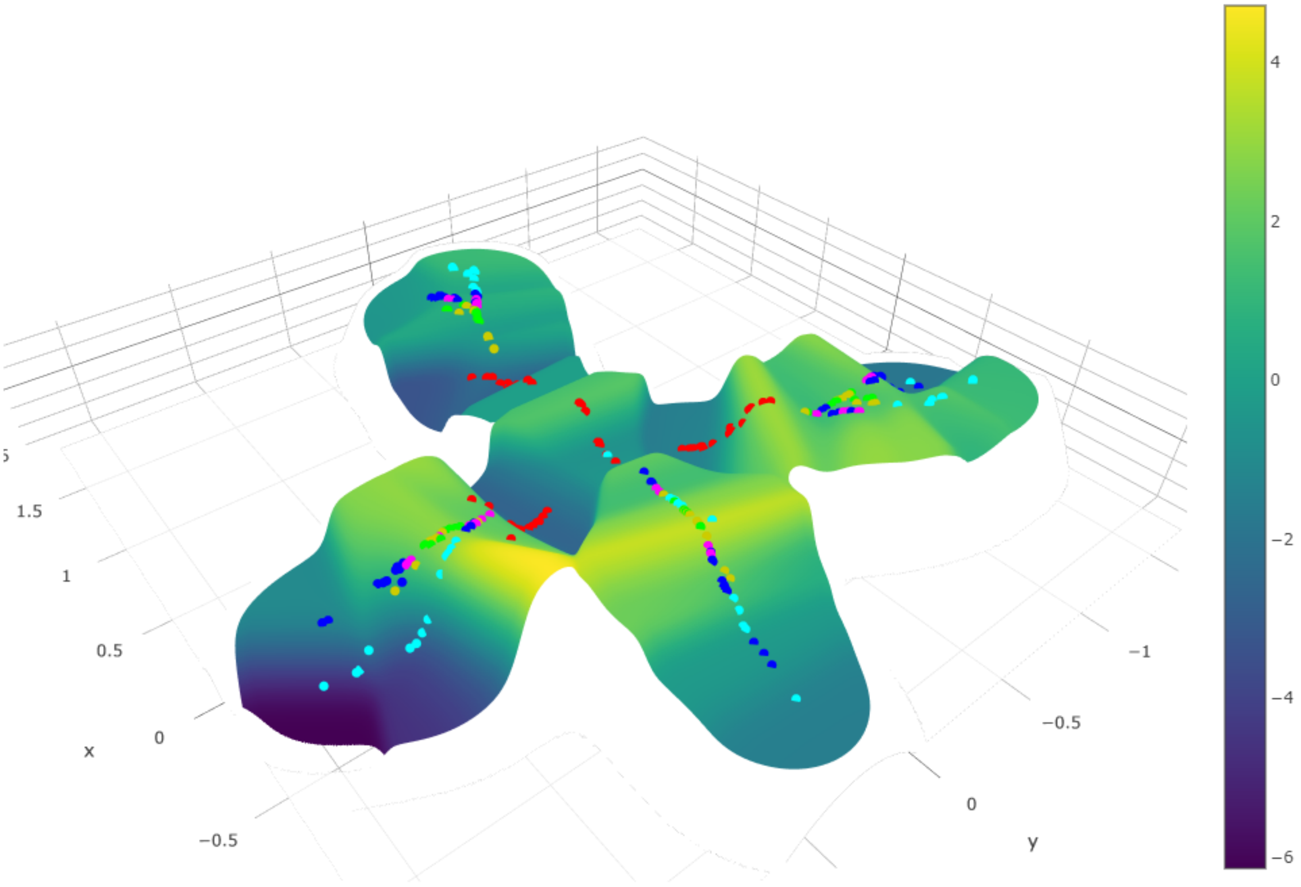

Supplementary Figure 4: Fitness Landscape visualization of the SynSyn data set. Samples are colored by mutagen (Red: 5-fluorouracil, Light green: amiloride, Blue: 5-azacytidine, Yellow: Mn^{2+}, Cyan: ribavirin, Magenta: mock).
