# Supplementary material for: DISSEQT—DIStribution-based modeling of SEQuence space Time dynamics
Source: Virus Evol. 2019 Aug 5;5(2):vez028. doi: 10.1093/ve/vez028 (PMC6680062; doi:10.1093/ve/vez028)
Supplement: vez028_Supplementary_Data [file vez028_supplementary_data.zip › SupplementaryMethods.pdf]

The Talus Plot

We will introduce the Talus Plot, a simple qualitative method for estimating the intrinsic dimension of a data set observed in noisy conditions. There already exists a plethora of methods for estimating the intrinsic dimension and one reason is that the problem is often inherently ill-posed. First, since the data set might be stretched out more along certain dimensions than others, the intrinsic dimension depends on the scale we are interested in. A narrow cylinder will for instance look like a line when zoomed out. Second, dimensions containing finer details of the data set might be masked or heavily corrupted by noise, making it impossible to make a clear-cut decision. The Talus Plot is designed to make it as easy as possible to find a dimension estimate that includes all dimensions that are detectable above the background noise. In particular, if the Talus plot gives a dimension estimate d, and the r ($r \leq d$) leading dimensions are removed, the new estimate will be $d - r$.

In the Talus Plot, $\log(\sigma_k) - \log(\sigma_{k+1})$ is plotted as a function of the dimension k, where σ_k is the k'th singular value of the data matrix. An example is shown in Figure 1. Clearly, the Talus Plot is a close relative to the Scree plot that displays σ_k^2 as a function of k. Taking the logarithm puts the singular values at a more reasonable scale where the background noise can be examined. As we will see, when noise dominates, the values in the Talus Plot will show small variations around a low mean. Thus, we find a dimension estimate using the Talus Plot by finding the breaking point at which the values start to exhibit this predictable behavior. To further understand the properties of the Talus Plot, we need some random matrix theory.

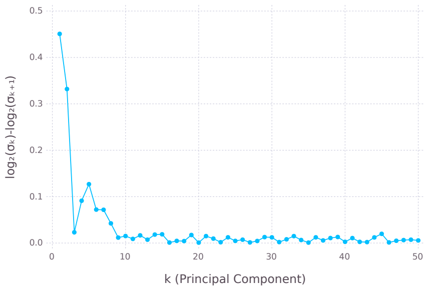

Figure 1: Talus Plot for a matrix X. The data matrix $X \in \mathbb{R}^{1000\times1000}$ is a rank 8 matrix with declining singular values with i.i.d. Gaussian noise added to each element. After $k = 8$, the Talus Plot shows small variation around a low mean, which is the expected behavior of a data matrix containing only noise.

Let $G(P, N)$ be the distribution of $P \times N$ random matrices with independent $\mathcal{N}(0, 1)$ elements. The distribution of the singular values of $A \sim G(P, N)$ (or equivalently, the eigenvalues of $A^T A$ since the k'th eigenvalue $\lambda_k = \sigma_k^2$) have been extensively studied in the literature [2]. The marginal distribution of unordered eigenvalues of $\frac{1}{P} A^T A$ converges asymptotically to the Marchenko-Pastur distribution [1] when $N, P \to \infty$ such that $\frac{N}{P} \to c \in [0, 1]$. The proba-

bility density function of the Marchenko-Pastur distribution is

$$f_{\mathrm{MP}}(x) = \frac{\sqrt{(c_+ - x)(x - c_-)}}{2\pi c x},$$

where $c_\pm = (1 \pm \sqrt{c})^2$, the domain of $f_{\mathrm{MP}}(x)$ is $[c_-, c_+]$ and we have assumed that $N \leq P$. The corresponding cumulative distribution function is

$$F_{\mathrm{MP}}(x) = \frac{1}{2} + x f_{\mathrm{MP}}(x) + \frac{(1+c)\arcsin\left(\frac{x-1-c}{2\sqrt{c}}\right) - (1-c)\arcsin\left(\frac{(1+c)x-(1-c)^2}{2x\sqrt{c}}\right)}{2\pi c},$$

which is shown in Figure 2 for some different values of c. Notice how taking the logarithm of the eigenvalues makes it possible to find a good approximation of F_{MP} using a simple linear model, at least for the upper half of the eigenvalues/singular values. The behavior of the rest of the eigenvalues will clearly not be of interest when detecting where the background noise starts to dominate. A few realizations are shown in Figure 3, exemplifying the linear trend.

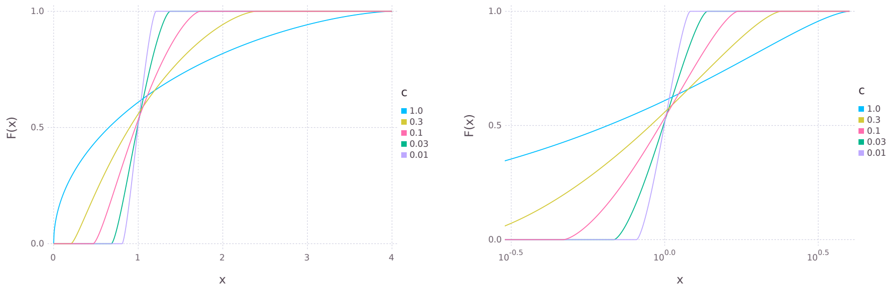

Figure 2: Cumulative distribution function of the Marchenko-Pastur distribution, for a few different values of c. Linear scale (left) and logarithmic scale (right) of the eigenvalues. Note that in the logarithmic scale, the CDF is well approximated by a linear function from the median ($y = 0.5$) and up, regardless of c.

The effects of the linear decline are clearly visible in the Talus Plots (see Figure 1), as the base level around which the values deviate. To understand the deviations, we will look at the random variables $\kappa_k := F_{\mathrm{MP}}(\lambda_k)$ that take values in $[0,1]$. Note that if one λ is randomly selected with equal probability from the set of random variables $\{\lambda_k;\ k = 1,\ldots,N\}$, then $F_{\mathrm{MP}}(\lambda)$ is by definition (asymptotically) uniformly distributed on $[0,1]$. The transformation has the effect of removing trends (linear or other), but keeping the dependence structure between the variables. The frequency contents of $\kappa_k - \kappa_{k+1}$ is shown in Figure 4, where the amplitude has been computed as the average over many realizations. Curiously close to a semi-circle, the amplitude peaks for the highest frequencies. Looking back at Figure 1, this corresponds well with the high-frequency oscillations that are visible in the singular values generated by the background noise.

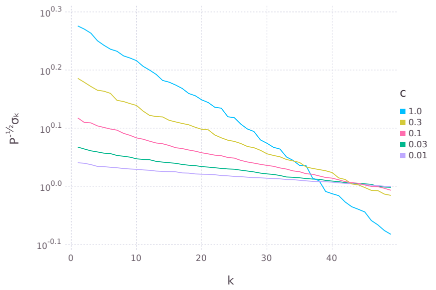

Figure 3: The singular values of A (scaled by $P^{-1/2}$), for a few different values of c. Each graph is a random realization. $A \sim G(P, N)$, $N = 99$ and $c = N/P$. The first 50 singular values are shown.

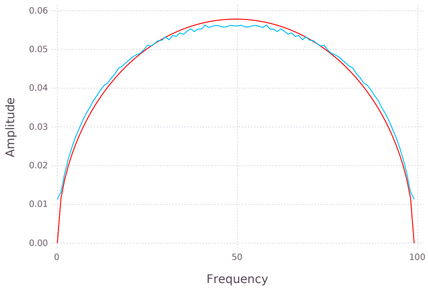

Figure 4: The absolute value of the coefficients in the discrete Fourier transform of $\kappa_k - \kappa_{k+1}$, computed as the average over 10000 random matrices. $P = 10000$ variables and $N = 1000$ samples. The shape is close to a semi-circle (red).

References

[1] Владимир А Марченко and Леонид А Пастур. “Распределение собственных значений в некоторых ансамблях случайных матриц”. In: *Математический сборник* 72.4 (1967), pp. 507–536.

[2] A. Edelman. “Eigenvalues and condition numbers of random matrices”. In: *SIAM Journal on Matrix Analysis and Applications* 9.4 (1988), pp. 543–560.
